# Shared responsibility for decision-making in NICU: A scoping review

**DOI:** 10.1177/09697330221134948

**Published:** 2023-01-23

**Authors:** Hanna-Kaisa Pellikka, Anna Axelin, Ulla Sankilampi, Mari Kangasniemi

**Affiliations:** 8058 University of Turku, Finland; 60654 University of Turku, Finland; 60650 Kuopio University Hospital, Finland; University of Eastern Finland, Finland; 60654 University of Turku, Finland; Satakunta Hospital District, Finland

**Keywords:** Decision-making, family-centred care, neonatal intensive care units, scoping review, shared responsibility

## Abstract

**Background:**

Shared responsibility is an essential part of family-centred care and it characterizes the relationship between parents and healthcare professionals. Despite this, little is known about their shared responsibility for decision-making in neonatal intensive care units.

**Aim:**

The aim of this scoping review was to identify previous studies on the subject and to summarize the knowledge that has been published so far.

**Method:**

The review was conducted using electronic searches in the CINAHL, PubMed, Scopus and PsycINFO databases and manual searches of the reference lists of the selected papers. The searches were limited to peer-reviewed papers that had been published in English from 2010 to September 2021. The data were selected based on inclusion and exclusion criteria and the findings were inductively summarized. We identified eight papers that met the inclusion criteria.

**Ethical considerations:**

The scoping review was conducted according to good scientific practice by respecting authorship and reporting the study processes accurately, honestly and transparently.

**Results:**

The results showed that shared responsibility for decision-making was based on the parents’ intentions, but the degree to which they were willing to take responsibility varied. The facilitating and inhibiting factors for shared responsibility for decision-making were related to the communication between parents and professionals. The impact was related to the parents’ emotions.

**Conclusion:**

It is essential that parents and professionals negotiate how both parties will contribute to their shared responsibility for decision-making. This will enable them to reach a mutual understanding of what is in the infants’ best interests and to mitigate the emotional burden of decisions in neonatal intensive care units. More research is needed to clarify the concept of shared responsibility for decision-making in this intensive care context.

## Introduction

Shared responsibility is an essential part of family-centred care, as it enables parents to help care for their child and share information and decision-making with healthcare professionals.^1^ The concept of shared responsibility has been identified as part of the general shared decision-making process between parents and healthcare professionals as nurses and physicians.^2^ Shared decision-making has been designed to enable parents and professionals to make decisions together^3^ and shared responsibility occurs when they accept mutual dependency. The parents need to engage in the process and be prepared to assume responsibility for their infant and the professionals need to be willing to share that care with them.^1^

Parents’ responsibilities have been linked to their infants’ right to well-being and this includes being cared for by their parents^4^ in hospitals.^5^ The nurturing relationship between parents and infants in neonatal intensive care units has been generally accepted as an essential quality improvement goal.^6,7^ Parents have been described as their infants’ primary caregivers, because they care for their infants and advocate for their needs, and this has been shown to promote well-being.^8^ Despite this, parents have reported that they found it stressful when their parental role was altered during their infants’ stay in a neonatal intensive care unit.^9^ This occurred when professionals took all the responsibility for the medical and nursing procedures,^10,11^ instead of educating the parents about how to participate in their infant’s care^10^ and helping them to adjust to their new role.^11^

The relationship between parents and professionals has been characterized by mutual dependency, where both stakeholders have expertise,^1,2,12^ and described as a power relationship.^1^ The partnership between parents and professionals changes during hospitalization, as parents gradually gain autonomy and control of their infant’s care.^1,12^ This has facilitated shared responsibility,^1,13^ and has provided professionals with the opportunity to enable parents to participate in caregiving and making decisions.^12^ Overall, the ideal decision-making process has been highlighted as a collaboration between parents and professionals,^14^ which involves shared responsibility.^1^

Previous studies have mainly found that parents’ participation in decision-making during hospitalization was a shared decision-making process,^2,15,16^ during situations such as serious illnesses^17^ and end-of-life decisions.^18^ Parents have said they felt responsible for decisions^19^ and wanted to take part in decisions that they regarded as part of their normal parental role.^20^ These included minor medical decisions.^21^ They have also seemed to prefer to focus on the big picture goals and delegate urgent and technical decisions to professionals.^20^ Hence, decision-making has been reported to rely on communication between parents and professionals^19^ about how to achieve their common goals with regard to the care^1^ and health of the infant.^2^

Little consideration has been given to what constitutes shared responsibility for decision-making in family-centred neonatal intensive care units. Shared responsibility is an important part of decision-making, because it can create mutual understanding about an infant’s care.^1,2^ In addition, shared responsibility plays a crucial role in enabling the safe and continuing transfer of responsibility from professionals to parents,^1,22^ during discharge and at home. This process supports the development of parenthood.^23^

## Aim

The aim of this study was to identify previous studies and summarize knowledge on the shared responsibility for decision-making between parents and professionals in neonatal intensive care units. The ultimate aim of this study was to increase knowledge and clarify the concept of shared responsibility for decision-making during neonatal intensive care. The review questions were:

1. What were the research methods used by the studies to examine shared responsibility for decision-making between parents and professionals?
2. What was the scope of each study and what were the concepts used to describe shared responsibility for decision-making between parents and professionals?
3. What findings did the studies report on shared responsibility for decision-making between parents and professionals?

## Method

A scoping review^24^ method was used, because it was suitable for examining how research was conducted and for clarifying concepts and identifying related factors.^25^ The review process consisted of five stages: identifying the research questions, identifying relevant papers, selecting papers for the review, charting the data and, finally, collating, summarizing and reporting the results.^24^

### Identifying the research questions

The first stage was to identify the research questions, based on the preliminary literature searches.^24^ The review questions were formulated to examine the scope of how the research was conducted,^25^ with regard to the research methods, the scope of the study and the concepts used. In addition, the third review question was posed to identify how shared responsibility was described in the literature.^25^ The aim of the first two review questions was to produce information that can be used for planning future research and the third question aimed to improve our understanding of the topic and identify any gaps in our knowledge.

### Identifying relevant papers

**Figure 1.**
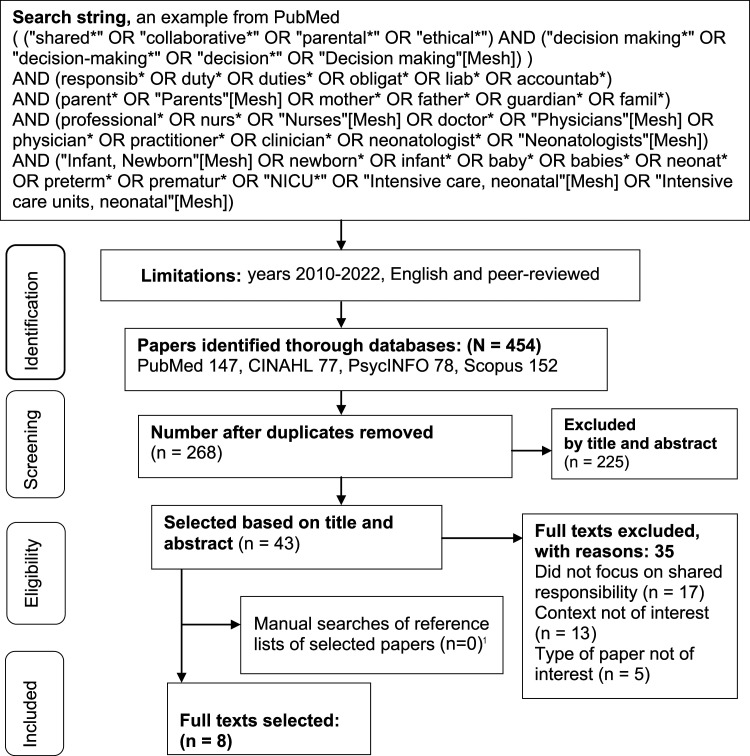
Flowchart of literature searches. ^1^Three were identified but they were among the eight papers that had already been selected.

The second stage was identifying relevant papers.^24^ Electronical searches were conducted using the PubMed, CINAHL, PsycINFO and Scopus databases (Figure 1). These were selected because they are considered relevant for healthcare related searches. The search terms consisted of free words and index terms, which were adapted according to the database being used. The searches were limited to peer-reviewed papers that were published in English from January 2010 to September 2022. This period was chosen to capture the latest knowledge on the study topic.^3^ Manual searches were carried out using the reference lists of the selected papers, with the same limitations. An information specialist checked the search strategy.

### Selecting papers for the review

During the third stage, the first author (H.-K. P.) selected the papers based on the inclusion and exclusion criteria.^24^ Papers were included if they focused on the responsibilities, or related concepts, for decision-making between parents and professionals. We also included papers that used empirical methods and discussion papers. The study participants had to include either parents or professionals, or both, and the professionals in this review were physicians and nurses. The context had to be, or include, care in a neonatal intensive care unit. We excluded papers that focused on shared responsibility between professionals or when the decision-making related to prenatal consultation or was made before delivery. Parental decisions about their infants’ participation in clinical research were also excluded.

The database identified 454 papers. These were uploaded to Mendeley and duplicates were removed (Figure 1), resulting in 268 papers. Then, the first author (H.-K. P.) used the inclusion and exclusion criteria to select 43 papers based on their titles and abstracts and eight based on their full text. The manual searches of the relevant reference lists identified three studies that met our criteria, but these had already been selected. This means that eight papers met the inclusion criteria and were included in this review (Table 1).

**Table 1.** Selected qualitative studies and discussion papers with aims, research methods, concepts and main results.

Selected studies	Aim of the study	Research methods	Used concepts	Main results
Qualitative studies				
Banazadeh et al.,^27^ Iran	To explore factors related to healthcare professionals that affected parents’ participation in decision-making for neonates with life-threatening conditions	Design: Qualitative study Scope of study: Parents’ participation in decision-making for neonates with life-threatening conditions Data collection: Semi-structured in-depth and face-to-face interviews Participants: 4 mothers and 6 fathers of hospitalized neonates, 4 nurses and 5 physicians Context: Neonatal intensive care unit Analysis method: Grounded theory	Responsibility, accountability	-Physicians feared being accountable to parents, which prevented them from allowing parents to participate in decision-making -Professionals abused their commitment to legal and professional responsibilities
Caeymaex et al.,^28^ France	To explore parents’ experience of the end-of-life decision-making process for their infant	Design: Qualitative study Scope of study: End-of-life decision-making process Data collection: Semi-structured face-to-face or telephone interviews Participants: 164 parents of 139 infants Context: Neonatal intensive care unit Analysis method: Discourse analysis	Responsibility, obligation	-Parents experienced emotional support, a trusting relationship and an explicit share of responsibility -Discussions and negotiations made it possible for parents to decide their role when making decisions -Shared responsibility for decision-making was clearly defined -Professionals supported parents’ participation in decision-making -Lack of discussions made parents accept the proposed role -Parents felt guilt and regret when they participated in life-and-death decisions -Parents felt involved and did not feel alone
Pellikka et al.,^29^ Finland	To describe parents’ perceptions of their responsibilities for their infant’s care during admission to a single-family room in a neonatal intensive care unit	Design: Qualitative study Scope of study: Parents’ responsibilities for their infant’s care Data collection: Semi-structured individual interviews Participants: 10 mothers and 9 fathers Context: Neonatal intensive care unit Analysis method: Inductive content analysis	Responsibility	-Parents knew their infant better than nurses -Shared responsibility was discussed and negotiated -Both parties had to know the limits of their responsibilities -Nurses were responsible for the safe implementation of care -Mutual trust led to shared responsibility
Shaw et al.,^30^ United Kingdom	Conversation analysis was used to examine the process of shared decision-making in the neonatal unit	Design: Qualitative study Scope of study: Shared decision-making process in conversations about redirection of care	Obligation, accountable	-Parents resisted making decisions when questions were about following recommendations
				-The parents’ burden was reduced when the doctor said that they were responsible for decisions
		Data collection: Formal conversations		
		Participants: 9 families and 6 physicians		
		Context: Neonatal intensive care unit		
		Analysis method: Conversation analysis		
Wright et al.,^31^ USA	To determine whether parents of critically ill premature infants felt that neonatal intensive care unit therapy was worthwhile, independent of their infant’s outcome	Design: Qualitative study	Responsibility	-Parents took their decision-making responsibilities seriously, because they wanted to promote their infant’s life and best interests
		Scope of study: Neonatal intensive care unit therapy		
		Data collection: Semi-structured and open-ended interviews		
		Participants: 10 mothers and 3 fathers		
		Context: Neonatal intensive care unit		
		Analysis method: Thematic analysis		
Discussion papers				
Dageville et al.,^32^ France	To propose a set of recommendations on the ethical principles to be respected in the making, and application of, end-of-life decisions	Design: Discussion paper Scope of study: End-of-life decisions Data: 35 references Context: Neonatal intensive care unit	Responsibility, obligation	-Legal constraints, moral obligations and medical responsibilities guided professionals -Laws restricted parents’ participation in end-of-life decisions
Foe et al.,^33^ Canada	To explore factors that appeared to contribute to both moral distress and the ‘moral schism’ for parents: The degree of available support, the coherence of the situation and a sense of responsibility	Design: Discussion paper Scope of study: Parents’ moral distress and the ‘moral schism’ related to decision-making Data: 24 references Context: Neonatal intensive care unit	Responsibility, duty	-No one person had the moral responsibility for decisions -Parents had a duty to their infant not to give up
Gillam and sullivan,^34^ Australia	To answer three questions. (1) What actually happens in practice: are parents involved in end-of-life decisions for their infants? (2) Do parents want to be involved in decision-making? (3) What effect does involvement in decision-making have on parents?	Design: Discussion paper Scope of study: Parents’ involvement in end-of-life decisions Data collection: 35 references, including 16 key references Context: Very young infants, including neonatal intensive care units	Responsibility, obligation	-Parents involvement varied from being included in discussions to making and taking responsibility for final decisions -Shared responsibility was related to guilt and regret

### Charting the data

The fourth stage was charting key information from the selected papers.^24^ Initially, the first author (H.-K. P.) read the papers several times to obtain an overview of the data. Then, the papers were tabulated according to the aim of the study, the methods, the concepts used and the main results (Table 1). In addition, the data for the third study question were extracted and organized as an Excel table.

### Collating, summarizing and reporting the results

**Figure 2.**
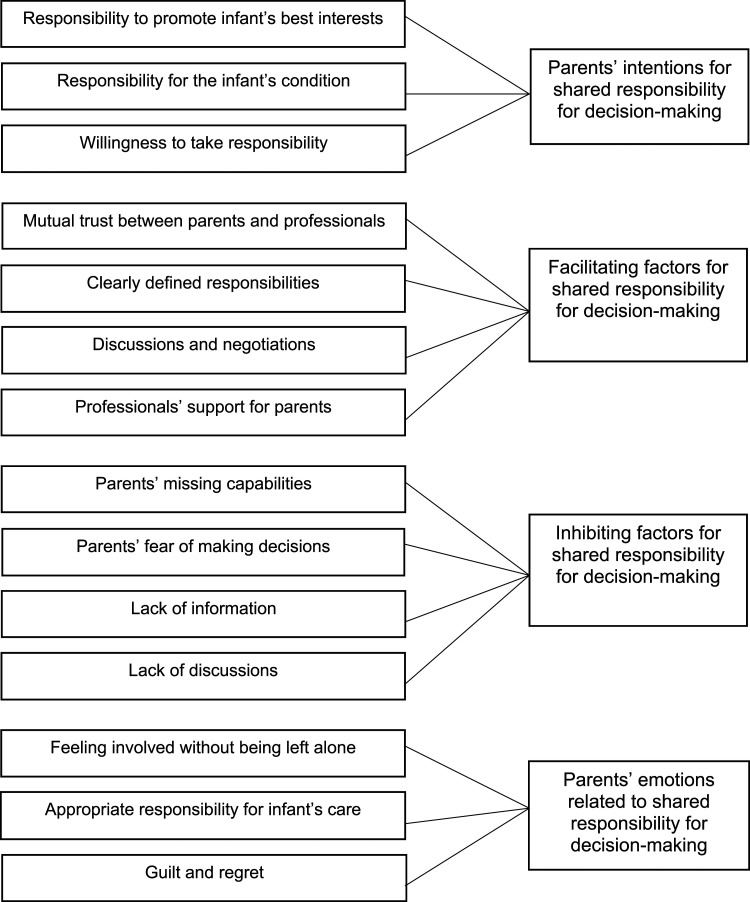
Shared responsibility for decision-making between parents and professionals.

The fifth stage was to collate and summarize the results.^24^ The first author (H.-K. P.) produced a narrative summary of the research methods, scope of the and the concepts used by the eight papers. In addition, the data regarding previous knowledge on the study topic was inductively analysed by grouping similarities and differences under descriptive themes with a higher abstraction level.^26^ (Figure 2).

## Results

### Research methods of previous studies

The research methods of the eight studies were identified and summarized according to the study design and data collection, participants, data analysis, context, year and country. The research methods are presented in a narrative form.

The study design in five of the eight papers was empirical, qualitative studies^27–31^ and three were discussion papers.^32–34^ Some of the data were collected with semi-structured interviews,^27–29,31^ including in-depth face-to-face,^27,28^ individual^28,29^ or couple interviews.^28^ One study used also non-structured telephone interviews.^28^ Other data were collected by observing decision-making situations^27^ and formal conversations between families and physicians.^30^

A total of 124 mothers, 79 fathers, 11 physicians and four nurses participated in the studies and there was one study that comprised nine families.^30^ The parents’ ages in one study ranged from 27 to 49 years and the healthcare professionals ranged from 35 to 59 years^27^ The mean age of the mothers was 34 in another study^28^ and one study stated that the mean age of the parents was 32.^29^ Some of the papers included the parents of infants who were born extremely preterm (before 28 weeks of gestation), very preterm (before 32 weeks),^27–31^ moderate to late preterm (32–36 weeks),^27–29^ and at full-term.^27,29–31^ The data collection took place when the infants were admitted to the neonatal intensive care units, which happened at 9–44 days of life in one study^27^ and 6 days to 8 months in another.^29^ One study focused on infants who were in a stable condition.^29^ Another focused on the discussions between parents and healthcare professionals about redirecting the care of critically ill infants.^30^ In one study, data was collected an average of 2.8 years after the infant’s death.^28^

The data analysis in the selected studies was conducted with grounded theory, discourse analysis,^27,28^ inductive content analysis,^29^ conversation analysis^30^ and thematic analysis.^31^ One paper used hermeneutics as a philosophical approach.^29^ The five empirical studies were conducted on neonatal intensive care units^27–31^ and one comprised single-family rooms.^29^ The discussion papers focused on neonatal intensive care units,^32,33^ although one also described wider facilities for very young infants.^34^ Two of the studies were carried out in France,^28,32^ with one each from Australia,^34^ Canada,^33^ England,^30^ Finland,^29^ Iran^27^ and the USA.^31^

### The scope of studies and the concepts used

The way that the scope of the studies was examined was divided into empirical studies and discussion papers. The concepts used for shared responsibility were studied from the point of view of the parents and the healthcare professionals.

The scope of the empirical studies focused on the parents’ participation in decisions about life-threatening^27^ and end-of-life scenarios^28^ and shared decision-making processes during conversations about the redirection of care.^30^ In addition, they focused on the parents’ responsibilities for their infant’s daily care^29^ and the reasons for initiating intensive care therapy.^31^ The discussion papers explored end-of-life decisions^34^ and recommendations on the ethical principles to be respected when making such decisions.^32^ They also examined the factors relating to both moral distress and moral schisms for parents in relation to the support parents received and their sense of coherence and responsibility.^33^

The concepts used for shared responsibility were responsibilities, obligations, duties and accountability. *Responsibility* referred to the parents’ ability to handle the implications of decisions they had made themselves or shared with professionals,^28,34^ and what they were expected to do for the sake of their infant.^29,31^ Responsibility was also investigated with regard to the consequences of the parents’ decision-making.^33^ From the professionals’ point of view, responsibility referred to their duty, based on ethical guidelines.^27,32^*Obligation* referred to the professionals’ responsibilities to act according to ethical and legal principles.^32^ Obligation referred to parents only having one choice in a situation^28^ or a strong bias towards a course of action.^30^ Obligation was also described as the parents’ duty to their infant.^34^*Duty* referred to the parents’ moral obligations and taking appropriate action.^33^*Accountability* referred to the duties of healthcare professionals,^27^ and *accountable* was used when professionals had to deal with the consequences that their actions had on the parents.^27,30^

### Previous knowledge on shared responsibility for decision-making

Inductive content analysis showed that shared responsibility for decision-making between parents and professionals was described in relation to the parents’ intentions and emotions and the factors that facilitated or inhibited the process (Figure 2). The deeper descriptions of the categories are presented.

#### Parents’ intentions regarding shared responsibility for decision-making

Parents were willing to promote their infant’s best interests and to avoid suffering.^31^ If their infants had been admitted to a single-family room, they felt responsible for participating in decision-making because they spent more time with their infant than the professionals and they were familiar with their infant’s condition.^29^ Parents said that the chance to participate in decision-making was right, as it enabled them to fulfil their obligations toward their infant.^34^ Although, parents were willing to participate in decision-making,^28,34^ their involvement varied from being included in discussions to making, and taking responsibility for, final end-of-life decisions.^34^

#### The facilitating factors for shared responsibility for decision-making

Shared responsibility for decision-making was facilitated by mutual trust between the parents and professionals^29^ and the parents trusted the doctors who guided them about decisions without placing pressure on them.^31^ Professionals respected the parents’ decisions and obligations to their infant and the values and beliefs related to them.^34^ Parents wanted their share of the responsibility for decision-making to be more clearly defined,^28^ so that both parties knew the limits of their responsibility.^29^ Discussions and negotiations between parents and professionals facilitated this.^29,34^ These discussions and negotiations allowed parents to ensure that they were comfortable with their degree of involvement in end-of-life decisions^34^ and other decisions regarding their infant’s care.^29^ Discussions also allowed parents to make daily care decisions and disagree when necessary.^29^

One facilitating factor for shared responsibility for decision-making was the support that the professionals gave the parents.^28,29^ Opportunities to explore alternative options helped parents to ask questions^30^ that they often feared^31^ and support from healthcare professionals included information that did not favour specific outcomes.^30^ When parents and professionals agreed, it provided the parents with comfort, security and protection against guilt when making end-of-life decisions.^28^ That support also strengthened the parents’ responsibility when they made autonomous decisions about daily care.^29^ The professionals’ legal constraints and moral obligations guided them to respect the parents’ autonomy and determine the infants’ best interests, according to their medical responsibilities.^32^ The parents perceived that, ultimately, the professionals had the final responsibility for implementing safe care.^29^

#### Inhibiting factors for shared responsibility for decision-making

Parents who were incapable of analysing situations regarding decisions,^28^ felt that the professionals were accountable for presenting things clearly.^30^ Consequently, parents were afraid to make medical decisions because they were concerned about the reasons for their decisions and this has affected their ability.^31^ Professionals neglected their professional and legal responsibilities and this prevented parents from participating in decision-making. They provided parents with limited information, in order to avoid questions, because they feared being accountable to them.^27^ Lack of explicit discussions often resulted in parents accepting the role given to them by the professionals when they made end-of-life decisions.^28^ They also reported feeling obliged to support their infant’s death when different professionals recommended this option or to agree verbally when they did not want to voice their decision out loud.^28^ It was also hard for parents to resist a course of action when the professionals used morally weighted recommendations for the redirection of care.^30^

#### Parents’ emotions related to shared responsibility for decision-making

Parents described feeling involved and were not left alone during end-of-life decisions.^28^ This was because the moral responsibility for decisions did not fall on one person when the decision was made by groups that included both parents and professionals.^33^ Parents felt relieved about that.^28^ Shared responsibility empowered parents to take appropriate responsibility, to be an equal part of the care team and to produce information on decision-making.^29^ It also offered an appropriate balance between active participation and being overwhelmed by the future weight of responsibilities.^28^

Emotions, such as guilt^28,34^ and regret about end-of-life decisions, were related to shared responsibility for decision-making,^34^ although one study reported that parents had no regrets.^31^ On the other hand, these feelings did not differ whether or not the parents took the final responsibility for decisions.^34^ The parents’ involvement in end-of-life decisions caused them internal conflict when they felt they had failed in their parental duty, which was often described as not giving up. However, they understood that the decisions were in their infants’ best interests.^33^ If the parents’ autonomous responsibility for life-and-death decisions went against their religious beliefs,^28^ the professionals framed the medical decisions as their own responsibility to avoid placing an emotional burden on the parents.^30^ In other words, the professionals made the decision and the parents gave their assent without feeling any responsibility.^28^

## Discussion

This scoping review identified previous studies and summarized knowledge on shared responsibility for decision-making between parents and professionals in neonatal intensive care units. The findings revealed the parents’ intentions and emotions related to shared responsibility for decision-making and the inhibiting and facilitating factors. However, there was an overall lack of knowledge in the existing literature and more research is clearly needed.

The parents’ intentions to take responsibility were a precondition for shared responsibility for decision-making. Family-centred care is a relatively new element of clinical practice in neonatal intensive care units^35^ and this change in care culture has enabled parents to share responsibilities and decision-making for their infants’ care with professionals.^1,10,13^ This important change has enabled parents to take responsibility for ensuring that their infants’ rights are respected^5^ and to be prepared for parenthood and the transition to family life at home.^23^ Shared responsibilities for decision-making between parents and professionals can support parents in their role and the family’s life at home after discharge.

Our findings showed that communication was both a facilitating and inhibiting factor for the process of shared responsibility for decision-making. This mirrors the communication challenges^16^ and implementation gaps^19^ that have also been reported in relation to shared decision-making. However, the communication between parents and healthcare professionals enabled them to understand their infants’ medical situation and engage in decisions about their treatment and care plans.^11^ Parents needed adequate knowledge to be able to make decisions^16^ and negotiate with professionals about their infant’s care.^12^ Trustful negotiations could prepare parents to take responsibility and enable them to find their desired level of shared responsibility. However, more empirical research is needed to explore, and describe, how parents and professionals negotiate, and define, their contribution for shared responsibility for decision-making. This should include the level of responsibility that parents want to adopt in different scenarios.

The findings showed when parents and professionals made decision together it decreased the moral stress on all parties, but there were also contrary findings. The literature stated that shared responsibility reduced parents’ feelings of responsibility for the outcomes of their decisions.^36^ Partnerships between parents and professionals were also described as protective, as they focused on mutual^1,2,12,16^ and shared goals that were in the best interests of the infants.^3,16^ However, parents said that their capacity or willingness to make decisions was affected when they faced overwhelming situations, especially end-of-life decisions. The parents depended on the authority and role of professionals to support them as parents,^16^ but the two parties were not equally responsible for decision-making in those situations. Parents preferred to make their own contributions about their infant’s best interest and for those to evolve during the infant’s admission. More research is needed on how shared responsibility evolves during hospitalization and how parents and professionals influence each other during this period.

Most of the papers we reviewed stated that the aim was to focus on decisions about medical care. In neonatal intensive care units, decisions about infants’ care included uncertainty about the potential long-term consequences.^3^ Our review found that end-of-life decisions affected parents emotionally, causing guilt and regret, even though they were not responsible for the final decision. These feelings of guilt have previously been reported^16,17^ in relation to parents’ reflections on the role they were expected to play for their infants.^18^ Feelings of guilt and regret have also been reported to effect parents’ decision-making and their ability to cope with the consequences.^17^ When parents make decisions for their infant, they have to balance the best interests of their infant and their own emotional burden.^3^ Thus, inappropriate moral responsibility can be avoided by acknowledging the parents’ willingness to take responsibility. On the other hand, parents said that it was important to participate in decisions that were linked to their normal role as parents^20^ and the related responsibilities.^19^ Future research is needed to explore, and describe, shared responsibilities between parents and professionals in different care situations, especially decisions about the infants’ daily care.

In this review, responsibility and its related concepts, obligations, duties and accountability were described as either as synonyms or very distinctive in relation to the decision-making process. Responsibility was mostly used to refer to parents’ individual responsibilities to participate in decision-making^28,34^ or professionals’ obligations to involve parents in the process.^27,32^ It is worth noting that responsibility was not the most prevailing concept in the studies regarding shared responsibility and shared responsibility was not included in the scope of the studies. However, shared responsibility for decision-making in family-centred care has been described as one of preconditions for mutual and successful outcomes for infant care.^1,2^ Our review showed that the concept of shared responsibility requires further investigation. Parents have the autonomy and control^2,12^ to carry out their responsibilities towards their infant and the care they have been asked to participate in. Shared responsibility with professionals enables parents to participate in their infants’ care in a safe and meaningful way. Thus, more research is needed to clarify how the concept of shared responsibility between parents and professionals can be defined.

### Strengths and limitations

The strengths of this review were linked to the method and data analysis. It was strengthened by using a scoping review method, because it allowed us to explore the concepts used in the selected studies and to combine different kinds of study methods.^24^ From a methodological point of view, any of the quantitative studies did not meet the inclusion criteria, thus the selected papers were qualitative studies, but the discussion papers were included to strengthen the review. The strength of the data analysis was that the first author (H.-K. P.) was responsible for the analysis process and the analysis was finalized by the research team.^37^

The limitations of the review concern the search strategy and the data extraction. The paper selection and data extraction were carried out by one researcher (H.-K. P.). This might have involved omitting relevant papers, but the researcher reduced this by double checking the review. In addition, some relevant citations might have been omitted during the data extraction phase.

### Ethical considerations

The review was conducted according to good scientific practice, by respecting other researchers’ work.^38^ The review process was also reported honestly and transparently.^39^

## Conclusion

This review highlights that shared responsibility can protect parents from harmful consequences and emotional distress when their infants are in neonatal intensive care units. Parents and professionals can achieve mutual understanding of an infant’s best interests and the meaningful participation of parents through negotiation and defining the parents’ contribution to shared responsibility for decision-making. In future, it is essential to clarify the concept of shared responsibility for decision-making, in order to promote the realization of family-centred care. This knowledge is needed to ensure safe and successful care delivery and the meaningful participation of parents in the care of their infant. More research is needed to clarify the concept of shared responsibility for decision-making in different kinds of care situations.
